# Enhancing Skeletal Muscle Rehabilitation: The Effects of Diclofenac Phonophoresis and Shock Wave Therapy on Serum Creatine Kinase in Athletes With Delayed-Onset Muscle Soreness

**DOI:** 10.7759/cureus.46267

**Published:** 2023-09-30

**Authors:** Selvaraj Sudhakar, Veena Kirthika S, Chanemougavally J, Shruthy K M

**Affiliations:** 1 Sports Physiotherapy, Faculty of Physiotherapy, Dr. M.G.R. Educational and Research Institute (Deemed to be University), Chennai, IND; 2 Physiotherapy (Neurology), Faculty of Physiotherapy, Dr. M.G.R. Educational and Research Institute (Deemed to be University), Chennai, IND; 3 Anatomy, A.C.S. Medical College and Hospital, Dr. M.G.R. Educational and Research Institute (Deemed to be University), Chennai, IND

**Keywords:** delayed-onset muscle soreness, skeletal muscle rehabilitation, creatine kinase, phonophoresis, shock wave therapy

## Abstract

Background

Delayed-onset muscle soreness (DOMS) is a common condition in athletes characterized by muscle pain and stiffness after intense or unfamiliar exercise. It significantly impairs an athlete’s performance by reducing muscle strength, flexibility, and overall physical capacity, often leading to suboptimal training or competition outcomes. Managing and mitigating DOMS is crucial for athletes to maintain peak performance and prevent potential injuries. The evaluation of effective treatment techniques is essential for expediting DOMS recovery by identifying biomarkers of skeletal muscle damage. This approach not only aids in optimizing recovery strategies but also contributes to the rehabilitation process, enabling athletes to return to peak performance quickly and safely. This study aims to evaluate the effects of shock wave therapy and diclofenac phonophoresis on serum creatine kinase levels in novice athletes suffering from DOMS in comparison to a control group. This research aims to assess the potential benefits of these therapeutic interventions in reducing skeletal muscle damage and enhancing recovery for individuals new to athletic training.

Methodology

A total of 48 novice athletes were recruited using simple random sampling and the block randomization approach to participate in this single-blind, multi-group, repeated-measures design. Male novice athletes in the age group of 18-25 years were included, and athletes with elevated serum creatine kinase variables at baseline measurements were excluded from the study. Before obtaining a written informed consent form, athletes were made aware of the procedure and associated risks. Group A received focused shock wave therapy, Group B received diclofenac phonophoresis, while Group C received no treatment. The Epley formula was used to compute the one-repetition maximum for each subject. Blood samples were taken at the baseline, 24, 48, 72, and 96 hours. Blood samples were taken before initiation of the study, as well as 24 hours, 48 hours, 72 hours, and 96 hours after DOMS was induced. Creatine kinase skeletal muscle biomarker was used as a dependent variable.

Results

In the evaluation of serum creatine kinase levels using analysis of variance (ANOVA), no statistically significant differences were observed between the three groups at baseline and 24-hour measurements (p > 0.05). However, statistically significant differences were found between the three groups at 48 hours, 72 hours, and 96 hours (p < 0.05). The repeated-measures ANOVA revealed significant overall changes in creatine kinase levels within the three groups, indicating time-dependent effects (p < 0.05). Specifically, during the 48 to 96-hour interval, the shock wave therapy group showed a lower mean value compared to the diclofenac phonophoresis group, followed by the control group.

Conclusions

The study demonstrates that a single administration of focused shock wave therapy effectively mitigated the elevation of creatine kinase levels in novice athletes with DOMS, surpassing the outcomes of diclofenac phonophoresis and the control group. These findings suggest the potential benefits of shock wave therapy in accelerating recovery from DOMS in the athletic population.

## Introduction

Delayed-onset muscular soreness (DOMS) is a type of muscular discomfort that develops after a person engages in a severe workout that requires prolonged contraction of the muscle. After eccentric activity, soreness usually starts to manifest eight to 24 hours later and peaks 48 to 72 hours later [1]. Most often, muscular soreness is felt after a muscle activity or when the muscle is palpated after rest. There is only a slight impression of muscular soreness, which typically goes away after 72 to 96 hours with only moderate soreness lasting over that point [2]. This exercise-related condition is referred to as diffuse-onset muscular soreness. DOMS is specifically recognized as one of the indications of muscle damage resulting from exercise involving eccentric contractions [3]. Athletes frequently encounter DOMS, especially after a prolonged break from training, due to modified training routines, or when starting a new program [4]. The repercussions of muscle soreness are diverse, ranging from mild-to-severe discomfort, hindrance to continuing with protective and effective training, heightened risk of injury due to abnormal mechanics, reduced muscle strength and power, decreased confidence, motivation, and willingness to engage in training sessions because of these negative effects [5]. Managing DOMS involves incorporating adequate warm-up and cool-down routines, gradual exercise progression, and implementing recovery strategies such as massage, foam rolling, and proper nutrition. While DOMS can be challenging, it is generally considered a natural response to intense physical activity and does not necessarily indicate significant harm to the muscles.

When skeletal muscle is injured, phosphocreatine in muscle tissue which is released into the circulation following muscle injury plays an important role in DOMS. High creatine kinase (CK) levels in the bloodstream suggest muscle damage after eccentric activity. CK is released as a result of muscle fiber breakdown, and microtrauma in the muscle causes muscular tightness and discomfort. Elevated CK levels are prevalent in individuals with DOMS when they participate in exercises that involve lengthening contractions [6]. Assessing CK levels can help determine the level of severity of DOMS and the degree of muscle damage induced by strenuous physical exercise. Skeletal muscle contains isoenzymes of CK discharged into the bloodstream with the onset of muscle pain. There are instances of creatine kinase-skeletal muscle (CK-MM) spikes identified predominantly in muscle tissue in individuals who have muscle injury with concentrations of intracellular molecules, indicating sarcolemma disruption after eccentric exercise [7].

Diclofenac is a non-steroidal anti-inflammatory drug known for its pain-relieving and anti-inflammatory properties. Ultrasound application includes the use of a coupling medium to promote the penetration of ultrasonic waves into bodily tissues. Ultrasonic waves stimulate diclofenac into the tissues through a process known as phonophoresis. This process aims to enhance the absorption of diclofenac gel into the skin, targeting inflamed muscle tissues. The effectiveness of diclofenac phonophoresis may depend on factors such as the concentration and formulation of the diclofenac gel, as well as the ultrasound parameters used during application. DOMS involves an inflammatory response in muscle tissues. Phonophoresis may facilitate the delivery of anti-inflammatory agents, which can help to reduce the inflammatory process and alleviate DOMS-associated symptoms. By targeting the specific area of muscle soreness with diclofenac, phonophoresis may contribute to a quicker recovery from DOMS [8]. Shock wave therapy has been investigated as a treatment for DOMS. This non-invasive therapy includes the application of high-energy sound waves to the afflicted muscle tissue to promote healing and decrease inflammation [9]. According to some studies, shock wave treatment may help decrease DOMS symptoms by increasing blood flow, tissue healing, and decreasing pain sensitivity. It is thought to hasten healing by boosting collagen formation and the release of growth factors [10,11]. However, the data is inconclusive, and further investigation is required to determine the efficacy of a single administration of focused shock wave treatment for DOMS. Thus, this study aimed to evaluate the effects of shockwave therapy and diclofenac phonophoresis on markers of muscle damage caused predominantly by eccentric contraction after exercise-induced muscle damage.

## Materials and methods

The study was designed as a pilot randomized clinical trial using a single-blind, multi-group, repeated-measures design [12]. The study was approved by the Institutional Ethical Committee of A.C.S. Medical College and Hospital, Velappanchavadi, Chennai, Tamil Nadu (reference number: ACSMCH/Ethical(43)/07-2018). The study followed the Declaration of Helsinki updated 2013 standards [13]. Using an alpha of 0.05, power of 0.8, and effect size of 0.47, a minimum sample size of 16 participants was determined using nMaster 2.0 power analysis software. Following the collection of demographic information, 48 novice athletes were chosen using simple random sampling and assigned using the block randomization approach. The research sample size was 16 per group to perform a pilot study that was appropriately powered [14]. Male novice athletes aged 18 to 25 years who were subjected to a specific training protocol for at least six months before participation were considered for inclusion in this study. Athletes with elevated serum CK at baseline measurements were excluded from the study [15].

Group A consisted of 16 subjects. A single administration of focused shock wave treatment (Storz Medical: Duolith SD1 T Top Ultra model equipment) was applied to the belly of the non-dominant biceps brachii muscle 24 hours after the induction of DOMS with treatment lasting approximately five minutes. Shock waves were generated by electrohydraulic mechanisms. The concentrated shock wave energy per unit area could vary from 0.06 to 0.09 mJ/mm^2^. The pulse ratio per point was 200. Subjects received 1,400 pulses with a total energy ranging between 10.3 and 15.4 mJ per point [10]. Diclofenac phonophoresis (Technomed Electroson 709 therapeutic ultrasound machine) was used on 16 subjects in Group B. The medicated phonophoresis coupling medium contained 1% diclofenac sodium topical gel and a non-steroidal anti-inflammatory drug for topical use. The approach was applied parallel to the non-dominant biceps brachii muscle at 24, 48, 72, and 96 hours following DOMS induction with parameters of 3 MHz frequency, pulsed mode, 1:4 ratio, and an intensity of 0.8 watts/cm^2^ for eight minutes [16,17]. Group C consisted of 16 subjects for whom no treatment was delivered and rest was advised. The Epley equation was adopted to determine the one repetition maximum (1RM) to the non-dominant elbow flexors for all subjects and an eccentric loading process of 80% 1RM was performed [18].

Measurement of creatine kinase

The International Federation of Clinical Chemistry (IFCC) defined a concept that adopts an updated recommended technique [19]. The Erba Chem 5 plus v2 equipment was utilized for the testing and 2 mL of blood samples were taken from all participants using a disposable syringe and serum was segregated. The serum was then diluted with 1 mL of CK reagent and maintained at 37°C for three minutes in an incubation chamber [20]. Agappe diagnostic devices were utilized for evaluation and values shown on the device were recorded. The study measured CK levels in novice athletes at baseline, 24 hours, 48 hours, 72 hours, and 96 hours after inducing DOMS. All participants remained in the study and their baseline parameters were within the normal range.

The data were analyzed using SPSS version 24.0 (IBM Corp., Armonk, NY, USA). The Shapiro-Wilk test was used to determine the normality of the data. Descriptive statistics with mean and standard deviation were reported. Analysis of variance (ANOVA) using repeated measurements analyzed within-group differences, whereas one-way ANOVA compared differences across groups. A p-value of less than 0.05 was considered significant.

## Results

**Figure 1:**
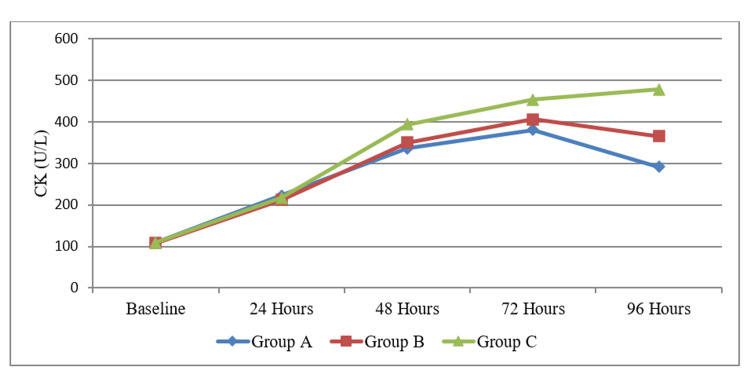
Graphical representation of creatine kinase levels among Group A, Group B, and Group C.

The demographic characteristics of novice athletes are shown in Table 1. In this study, 48 novice athletes were chosen as participants and divided into three groups. Group A underwent shock wave treatment, Group B received diclofenac phonophoresis, and Group C was the control group. A one-way ANOVA test was used to analyze the mean values of CK levels among three groups. The findings revealed that at the baseline and 24-hour measurements, there were no significant differences in CK levels among the three groups (p > 0.05). However, after 48 hours, 72 hours, and 96 hours, there were statistically significant differences between the groups (p < 0.05). Table 2 represents the outcome of the CK levels using the one-way ANOVA test between the three groups, and Figure 1 provides a graphical representation of the CK levels between the three groups.

**Table 1:** Demographic characteristics of the subjects recruited in Group A, Group B, and Group C.

Parameters	Group A	Group B	Group C	P-value
N	16	16	16	
Age	20.4 ± 2.1	20.6 ± 2.8	21.1± 2.8	0.71
Height (cm)	163 ± 3.2	162 ± 3.9	162 ± 3.5	0.78
Weight (kg)	61.5 ± 2.9	61.1 ± 3.2	61.9 ± 2.7	0.89
Body mass index (kg/m^2^)	23.20 ± 2.7	23.32 ± 2.4	23.62 ± 1.9	0.94

**Table 2:** Comparison of creatine kinase levels among Group A, Group B, and Group C. *: P-values >0.05 are not significant; **: P-values <0.05 are significant. CK: creatine kinase; ANOVA: analysis of variance

Variable CK (U/L)	Group A		Group B		Group C		ANOVA F value	Significance
	Mean	SD	Mean	SD	Mean	SD		
Baseline	109.56	12.88	108.00	10.23	109.37	10.53	0.058	0.944*
24 hours	223.25	34.27	212.37	25.02	217.37	32.39	0.499	0.611*
48 hours	336.06	36.08	350.00	27.63	394.93	40.31	12.31	0.000**
72 hours	381.18	45.49	406.87	29.85	453.93	40.75	14.13	0.000**
96 hours	292.00	35.32	365.37	31.06	478.81	39.50	112.67	0.000**

## Discussion

The primary goal of this study was to determine the efficacy of a single administration of focused shock wave treatment and diclofenac phonophoresis in identifying the biochemical indicators of DOMS. The condition was chosen as the investigative framework of this study in novice athletes with an appropriately fundamental design. Experiencing severe muscle soreness can lead an individual to exert a higher force than they are normally accustomed to. For instance, if someone is prescribed to work at a specific level of their 1RM, they may need to train at a predetermined intensity even during the presence of DOMS. Moreover, changes in the strength ratio between agonist and antagonist muscle groups, influenced by biochemical variations, could result in an increased occurrence of skeletal muscle damage. Shock wave therapy for DOMS is used to potentially alleviate the symptoms and promote faster recovery.

Shock wave therapy has stimulated the formation of new blood vessels which improve blood flow and oxygen supply to the muscle tissue, promoting rich nutrient delivery and waste removal. Enhanced blood supply aids in tissue repair and regeneration. Shock waves trigger the release of transforming growth factor-beta and vascular endothelial growth factor. These growth factors stimulate cell proliferation, help in tissue repair processes, and induce a controlled inflammatory response in the treated area. This can lead to the release of various cytokines and chemokines, which play essential roles in tissue healing and regeneration. Collagen is a critical protein that provides structural support to muscle tissue. Shock wave therapy has been shown to enhance collagen production, which can contribute to the healing of damaged muscle fibers and improve tissue integrity [21].

Shock wave therapy has been studied for its potential effects on biochemical markers of muscle damage. CK is an enzyme present in muscle cells that is typically elevated in the blood as an indication of muscle injury. Shock wave therapy temporarily raises CK levels, indicating that the treatment induces controlled muscle damage. This transient increase is believed to trigger the body’s natural healing response, resulting in tissue repair and regeneration as well as shock wave treatment has the potential to cause a local inflammatory reaction in the treated region [22]. Pro-inflammatory cytokines such as interleukin-6 and tumor necrosis factor-alpha are released during this inflammatory response. These cytokines play an essential role in the initial phases of tissue repair and regeneration. Shock wave treatment, in addition to pro-inflammatory cytokines, increases the production of anti-inflammatory cytokines such as interleukin-10. These cytokines aid in the regulation of the inflammatory response, limiting over-inflammation and encouraging a balanced healing process [23].

Ultrasound waves can improve the absorption and penetration of diclofenac into the deeper layers of muscles, potentially increasing its effectiveness in reducing inflammation and pain associated with DOMS. The combined therapy allows for targeted application, focusing on the specific area affected by DOMS. This may lead to more efficient pain relief and a reduction in inflammation. The synergistic effects of ultrasound and diclofenac could contribute to a quicker recovery from DOMS, allowing individuals to resume their activities with less discomfort. Diclofenac can provide analgesic effects by inhibiting the production of prostaglandins, which are chemicals involved in the sensation of pain. By reducing prostaglandin levels, diclofenac may help alleviate DOMS-related pain [24].

Ultrasound therapy has anti-inflammatory benefits, which help minimize muscular inflammation, and as a result, may have an influence on CK levels. By controlling inflammation, it aids in healing and reduces CK levels over time. Ultrasound therapy increases blood flow to the treated region, which may assist in the supply of nutrients and oxygen to the muscles. Improved blood circulation aids in the elimination of waste product CK from the affected muscles. Ultrasound treatment has been shown to aid in tissue repair and regeneration. Phonoporesis accelerates the healing process after muscle damage and leads to a faster normalization of CK levels [25,26].

Before the initiation of DOMS, the baseline CK values of all three groups remained within the same range. However, following the induction of DOMS, CK levels began to rise in all three groups. Immediately after therapy, there was a difference in recovery in relation to CK in all three groups. At baseline and 24 hours, CK levels did not differ substantially across the three groups (p > 0.05). However, significant changes were identified at 48 hours, 72 hours, and 96 hours (p < 0.05). The study revealed that diclofenac phonophoresis has a minor effect in lowering CK levels. Shock wave treatment, on the other hand, has a greater impact on lowering CK activity. This suggests that the shock wave treatment utilized in this study minimizes biochemical changes in the skeletal muscle caused by unfamiliar and intense eccentric activity. Thus, the rise in plasma CK activity was substantially larger in the initial phase followed by a significant reduction in CK level in the shock wave group compared to the control and diclofenac phonophoresis groups.

Study limitations

This research conducted a single-centered, single-blinded study involving male novice athletes aged 18 to 25 years. The study induced DOMS in the non-dominant elbow flexor muscles and assessed muscle damage using CK because direct damage assessment through muscle biopsies was not feasible. The study did not extensively explore the correlation between body mass index and muscle damage biomarkers. Additionally, the study did not account for various factors such as environmental influences, climate variations, and subjects’ hydration status.

## Conclusions

A single administration of focused shock wave treatment considerably reduced the rise of CK levels when compared to diclofenac phonophoresis and the control group. Based on these data, it is possible to infer that shock wave treatment is an effective strategy for reducing CK levels following eccentric activity. Shock wave therapy has the potential to accelerate the regeneration process, allowing novice competitors to return to competing in sports sooner.
